# Fabrication and Characterization of CMOS-MEMS Thermoelectric Micro Generators

**DOI:** 10.3390/s100201315

**Published:** 2010-02-09

**Authors:** Pin-Hsu Kao, Po-Jen Shih, Ching-Liang Dai, Mao-Chen Liu

**Affiliations:** 1 Department of Mechanical Engineering, National Chung Hsing University, Taichung, 402, Taiwan; E-Mails: d9461402@mail.nchu.edu.tw (P.-H.K.); d9361301@mail.nchu.edu.tw (M.-C.L.); 2 Department of Civil and Environmental Engineering, National University of Kaohsiung, Kaohsiung, 811, Taiwan; E-Mail: pjshih@nuk.edu.tw

**Keywords:** thermocouples, micro generators, CMOS-MEMS

## Abstract

This work presents a thermoelectric micro generator fabricated by the commercial 0.35 μm complementary metal oxide semiconductor (CMOS) process and the post-CMOS process. The micro generator is composed of 24 thermocouples in series. Each thermocouple is constructed by p-type and n-type polysilicon strips. The output power of the generator depends on the temperature difference between the hot and cold parts in the thermocouples. In order to prevent heat-receiving in the cold part in the thermocouples, the cold part is covered with a silicon dioxide layer with low thermal conductivity to insulate the heat source. The hot part of the thermocouples is suspended and connected to an aluminum plate, to increases the heat-receiving area in the hot part. The generator requires a post-CMOS process to release the suspended structures. The post-CMOS process uses an anisotropic dry etching to remove the oxide sacrificial layer and an isotropic dry etching to etch the silicon substrate. Experimental results show that the micro generator has an output voltage of 67 μV at the temperature difference of 1 K.

## 1. Introduction

With the development of electronic production processes, consumer electronic devices have become smaller and more portable. Power is an important issue for electronic devices. Micro generators can be applied in electronic devices providing additional power. The function of micro generators is not only to produce electric power but also to recycle and reuse waste energy. Many kinds of energy can be converted into electric power, for instance, wind power [1], thermal power [2], water power [3], chemical energy [4], and nuclear energy [5]. However, some energies cause environmental pollution and are not stable sources. Thermoelectric micro generators have a potential to convert heat into electric power. The heat is generated when electronic products are operating, so thermoelectric micro generators can recycle the heat as electric power and can reduce thermal spreading and alleviate global warming. Several micro devices have been manufactured using microelectromechanical system (MEMS) technology [6]. Many studies have employed MEMS technology to develop thermoelectric generators. For example, Huesgen *et al.* [7] used a combined surface and bulk micromachining process to fabricate thermoelectric generators with thermocouples on the silicon wafer. One thermocouple junction was connected by electroplated metal strips to the heat source and insulated to the heat sink by a cavity in the substrate. Wang *et al.* [8] proposed a thermoelectric micro generator based on n-type and p-type Bi2Te3 nanowire array thermoelectric materials. The nanowire arrays were produced using electrochemical deposition of Bi2Te3 on the nano-pores of an alumina template, and the Seebeck coefficient of p-type and n-type Bi2Te3 nanowire arrays was about 260 and −188 μV/K, respectively. Sato *et al.* [9] manufactured a thermoelectric generator using the thick-film formation processes with gold electroplating and deep reactive ion etching of silicon. The thermoelectric generator of gold and silicon produced voltages in accordance with the temperature difference at the thermocouples. Qu et al. [10] presented a flexible thermoelectric generator that’s fabrication involved foil lithography, electroplating, embedding and wet chemical etching. The generator was composed of Sb-Bi thermocouple strips embedded in a 50 μm thick flexible epoxy film and was capable of generating a voltage of 0.25 V at a temperature difference of 30 K. If the fabrication of thermoelectric generators is compatible with the commercial CMOS process, then the generators have a potential to combine with integrated circuits on a chip. The processes of the above studies [6–9] are not compatible with the CMOS process. In this study, we develop a thermoelectric generator that’s fabrication is compatible with the CMOS process. Thermocouples, which are able to convert heat into voltage output, can be designed as a temperature sensor or a thermoelectric generator. The difference between both the devices is that the output of a thermoelectric generator focuses on the output power besides the output voltage. In this work, thermocouples are adopted to design the thermoelectric micro generator.

The use of the commercial CMOS process to manufacture MEMS devices is called CMOS-MEMS technique [11–13]. Micro devices fabricated by the CMOS-MEMS technique are usually a post-CMOS process to release the suspended structures [14] or to coat the functional films [15]. The advantages of CMOS-MEMS micro devices include compatibility with integrated circuits, low cost per unit area, and mass-production utilizing semiconductor foundries. In this work, we employ the CMOS-MEMS technique to fabricate a thermoelectric micro generator. The structure of the micro generator consists of 24 thermocouples in series and an aluminum plate, and the hot part of the thermocouples is connected to the aluminum plate that can conduct heat and increase the heat-receiving of the hot part. The cold part of the thermocouples is covered with a silicon dioxide layer for insulating the heat source. In order to increase the temperature difference at the thermocouples, the aluminum plate and the hot part of the thermocouples are designed as suspended structures. Thereby, the micro generator needs a post-CMOS process to release the suspended structures. The post-CMOS process includes an anisotropic dry etching to etch the silicon dioxide layer and an isotropic dry etching to remove the silicon substrate under the suspended structures. The experimental results depict that the output voltage of the micro generator is 67 μV at the temperature difference of 1 K.

## 2. Design of the Generator

**Figure 1.**
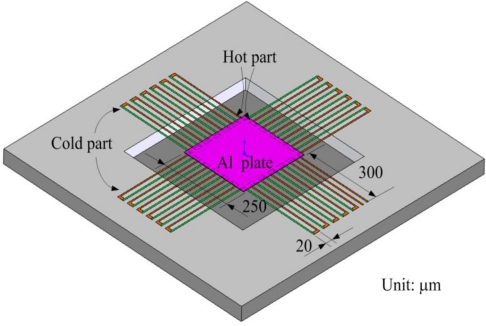
Schematic of the thermoelectric micro generator.

Figure 1 illustrates a schematic of the thermoelectric micro generator. The generator consists of 24 thermocouples in series. The materials of the thermocouples are n-type and p-type polysilicons. Each thermocouple is constructed by two strips; one strip is p-type polysilicon and the other is n-type polysilicon. The dimensions of each thermocouple are 640 μm long, 5 μm wide and 0.3 μm thick. The area of the generator is about 850 × 850 μm^2^.

**Figure 2.**
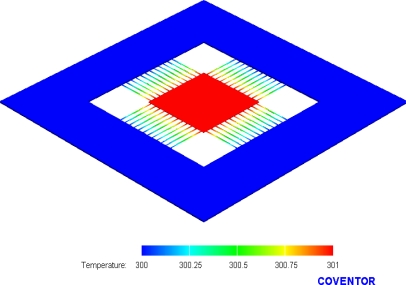
Simulation of temperature distribution for the generator.

**Figure 3.**
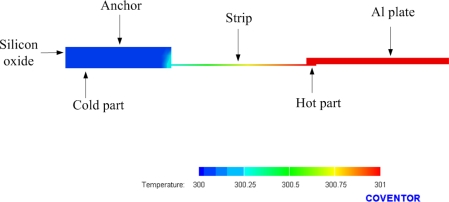
Cross-sectional view of temperature distribution for the generator.

As shown in Figure 1, one junction of p-type and n-type polysilicon strips covered with silicon dioxide is the cold part of the thermocouples, and the other junction of p-type and n-type polysilicon strips to be suspended is the hot part of the thermocouples. In order to increase the temperature difference between the hot and cold parts, silicon dioxide with low thermal conductivity is utilized to cover the cold part. In addition to being suspended, the hot part is connected to an aluminum plate, which is used to conduct heat and increase the heat-receiving area. The finite element method (FEM) software, CoventorWare, is employed to simulate the temperature distribution of the micro generator. The model of the generator was established in accordance with Figure 1, and the Manhattan brick was used to mesh the model. The parabolic element was adopted. The thickness and thermal conductivity of the materials are summarized in Table 1. The initial temperature of 300 K was set, and the heat source of 301 K was applied to the aluminum plate of the generator. Figure 2 shows the simulated results of temperature distribution for the micro generator. The results showed that the suspended aluminum plate had a uniform temperature of 301 K. Figure 3 illustrates the cross-sectional temperature distribution for half of the micro generator. The silicon dioxide layer over the cold part of the thermocouples successfully isolates the heat source.

**Figure 4.**
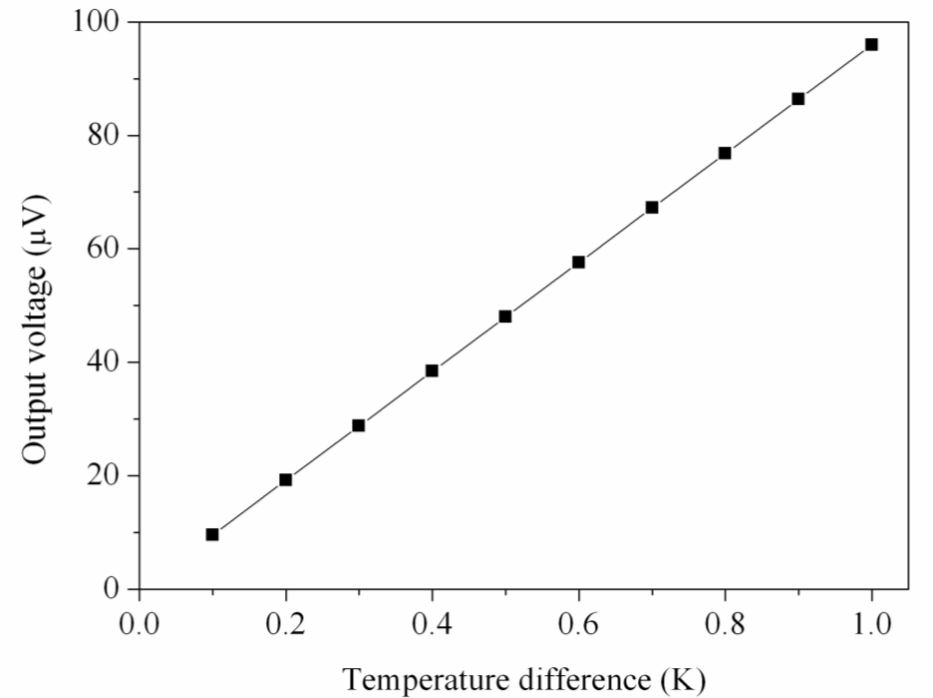
Simulated results of the output voltage for the generator.

The output voltage depends on the number of thermocouples. The relationship between the output voltage and the number of thermocouples is given by [16],

(1) $$V_{\mathrm{out}}=n(\alpha_{1}-\alpha_{2})(T_{h}-T_{c})$$

where *V_out_* represents the output voltage generated by the thermoelectric effect; *n* is the number of thermocouples connected in series; *α_1_* and α_2_ are the Seebeck coefficients of p-type and n-type polysilicons, respectively; and *T_h_* and *T_c_* are the temperatures of the hot and cold parts, respectively. According to Equation (1), we know that the output voltage relies on the number of thermocouples, the Seebeck coefficient of materials and the temperature difference. The output voltage, *V_out_*, is proportional to the number of thermocouples, *n*, and the relative Seebeck coefficient, *α_1_ – α_2_*. In this work, the n-type and p-type polysilicons are adopted as the materials of the thermocouples owing to their large relative Seebeck coefficient. In this design, the number of thermocouples is 24, and the relative Seebeck coefficient of the n-type and p-type polysilicons is about 0.004 mV/K. The output voltage of the generator can be evaluated by substituting the values *n* = 24 and *α_1_ – α_2_* = 0.004 mV/K into Equation (1), and the results are plotted in Figure 4. The calculated results show that the output voltage of the generator was 96 μV at the temperature difference of 1 K.

If the external load, *R*, that is the same the inertial load is connected, the maximum output power of the generator can be obtained by [9],

(2) $$P_{\text{max}}=\frac{V_{\mathrm{out}}^{2}}{4R}$$

Furthermore, substituting Equation (1) into Equation (2), the maximum output power of the generator can be written as,

(3) $$P_{\text{max}}=\frac{n^{2}{\left(\alpha_{1}-\alpha_{2}\right)}^{2}{\left(T_{h}-T_{c}\right)}^{2}}{4R}$$

**Figure 5.**
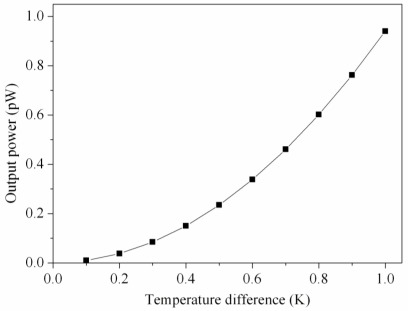
Simulated results of the output power for the generator.

In this design, the parameters are *n* = 24, *α_1_ – α_2_* = 0.004 mV/K and *R* = 2.45 kΩ. Substituting the values into Equation (3), the maximum output power of the generator can be yielded and the results are plotted in Figure 5. The results show that the output power of the generator was about 0.94 pW at the temperature difference of 1 K.

## 3. Fabrication of the Generator

**Figure 6.**
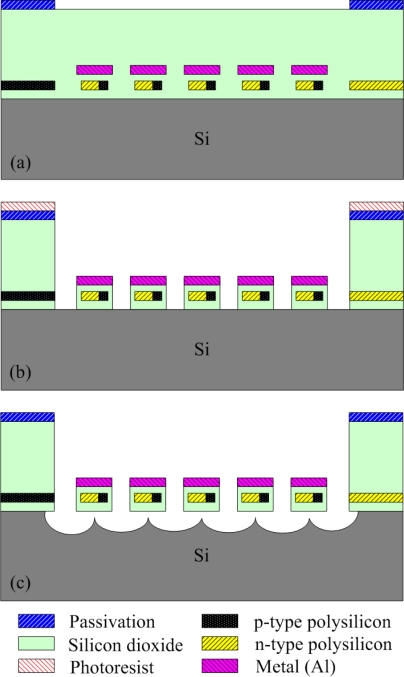
Process flow of the generator (a) after completion of the CMOS process; (b) etching the oxide sacrificial layer; (c) etching the silicon substrate.

**Figure 7.**
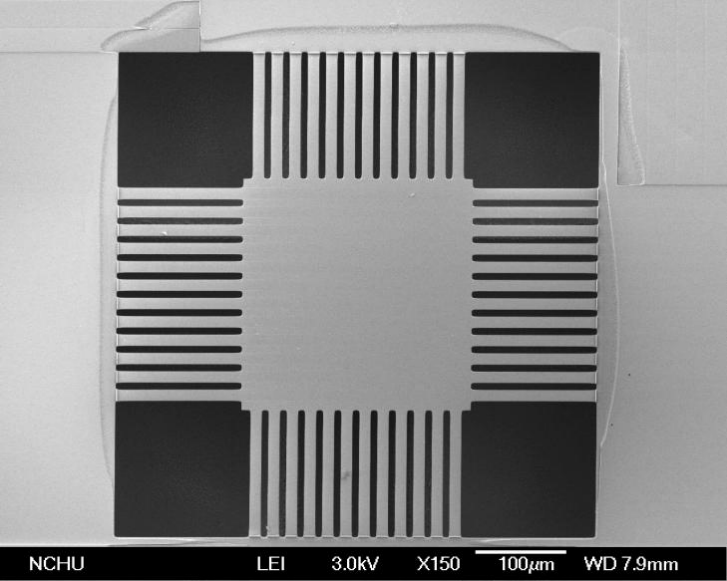
SEM image of the generator after the post-CMOS process.

The thermoelectric micro generator is manufactured using the commercial 0.35 μm CMOS process of Taiwan Semiconductor Manufacturing Company (TSMC). Figure 6 illustrates the process flow of the micro generator. Figure 6a shows the cross-section of the micro generator after completion of the CMOS process. The thickness of the sacrificial oxide, field oxide, polysilicon, aluminum, and passivation layers is about 6.4 μm, 0.28 μm, 0.3 μm, 0.6 μm and 1 μm, respectively. In order to insulate the heat sink of the hot part and the aluminum plate, these structures have to be suspended, so the generator needs a post-CMOS process to etch the sacrificial layers, and to release the suspended structures. The post-CMOS process contains three steps. First, a photoresist is coated on the surface of the generator using a spinner, and it is patterned by a lithograph. Then, the oxide sacrificial layer is etched using a reactive ion etch (RIE) with CHF_3_/O_2_, which is an anisotropic dry etching for the oxide layer. Figure 6b displays the oxide sacrificial layer in the generator to be removed after the anisotropic dry etching. Next, the silicon substrate is etched using a RIE with XeF_2_, which is an isotopic etching for the silicon substrate, so that the suspended structures are released owing to undercut. Figure 6c shows the suspended structures released after the isotropic dry etching. Finally, the photoresist is striped by acetone. Figure 7 presents a scanning electron microscope (SEM) image of the micro generator after the post-CMOS process.

## 4. Results and Discussion

**Figure 8.**
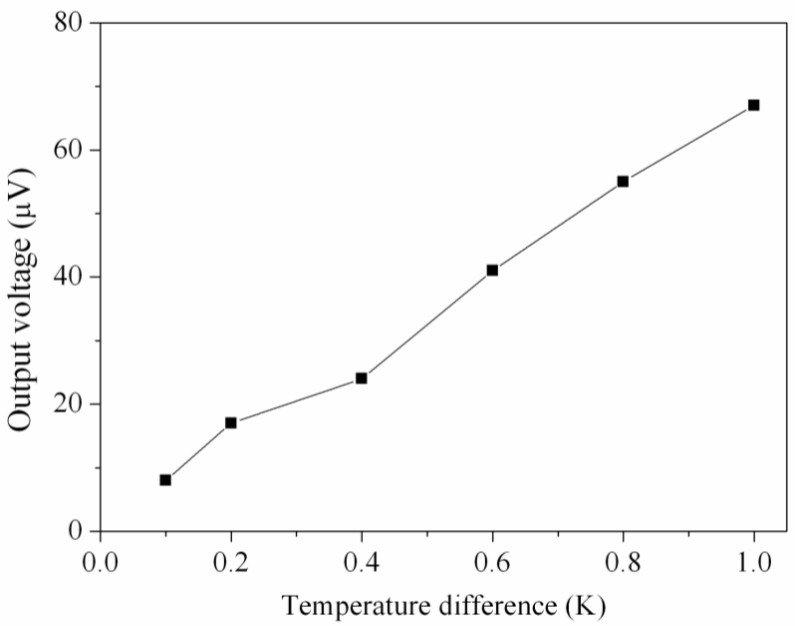
Output voltage of the generator.

A heat source, an infrared temperature detector, and a multifunction electrical meter were utilized to measure the characteristics of the micro generator. The heat source provided a heat flux to the micro generator, and the infrared temperature detector was used to measure the temperature difference between the hot and cold parts of the thermocouples. The multifunction electrical meter was employed to measure the output voltage of the generator. Figure 8 depicts the measured results of the output voltage for the micro generator. The measured results show that the generator had an output voltage of 41 μV at the temperature difference of 0.6 K, and the output voltage was 67 μV at the temperature difference of 1 K. The output voltage per area of the generator was about 0.093 mV mm^−2^ K^−1^. Compared with the simulated results in Figure 4, where the simulated output voltage was 96 μV at the temperature difference of 1 K, the percentage of error is about 30%. The cause of the error was either that the Seebeck coefficient of the materials in the thermocouples was changed by variations in the fabrication process or that the infrared temperature detector was not precise enough.

**Figure 9.**
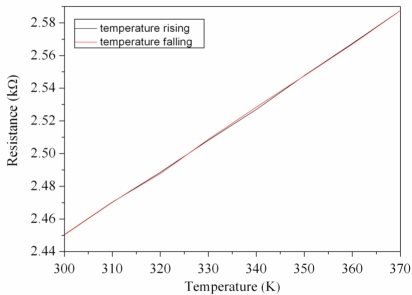
Resistance of the generator.

In addition, the resistance of the micro generator was detected. The heat source supplied a heat flux to the micro generator, and the resistance of the generator was measured by the multifunction electrical meter. Figure 9 shows the change of resistance for the generator with the temperature. The experimental results show that the resistance of the generator varied from 2.45 to 2.59 kΩ as the temperature changed from 300 to 370 K. The resistance of the generator was proportional to the temperature, and it recovered to the initial state after the temperature fell, which the paths in Figure 9 were almost the same.

**Figure 10.**
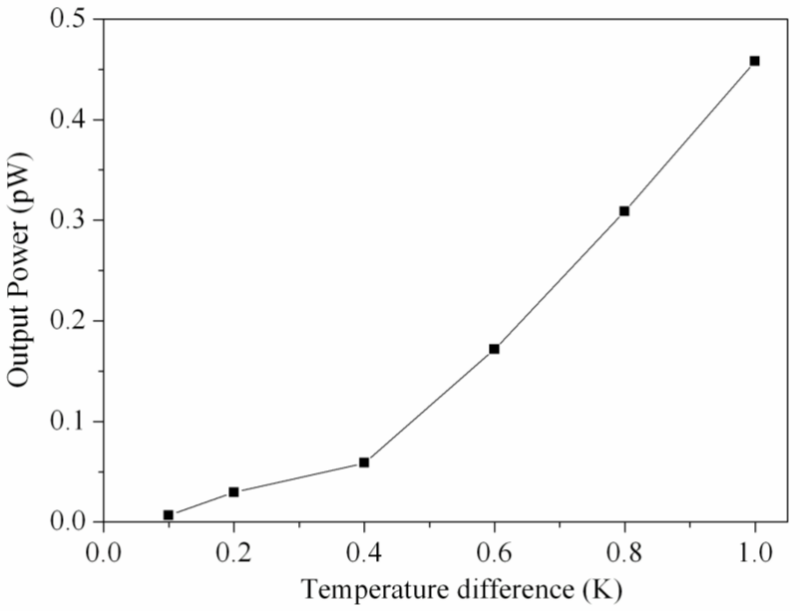
Output power of the generator.

According to Equation (2), the output power of the generator relies on its output voltage and resistance. The measured output voltage in Figure 9 and the resistance *R* = 2.45 kΩ were substituted into Equation (2), the maximum output power of the generator was obtained, and the results were shown in Figure 10. The results revealed that the generator had an output power of 0.16 pW as the temperature difference of 0.6 K, and the output power of the generator increased to 0.46 pW as the temperature difference changed to 1 K. The power factor [7] of the generator was 6.4 × 10^−7^ μW mm^−2^ K^−2^. A comparison to the simulated results in Figure 5, the simulated output power was 0.94 pW at the temperature difference of 1 K, so the output power had an error of 0.48 pW at the temperature difference of 1 K. Because the measured output voltage had an error existence, the output power in Figure 10 was evaluated by the measured output voltage, leading to the error in the output power.

Huesgen *et al.* [7] presented a thermoelectric micro generator with thermocouples made of Al and n-poly-Si, and it had an output voltage per area of 7.46 mV mm^−2^ K^−1^ and a power factor of 1.612 × 10^−4^ μW mm^−2^ K^−2^. Strasser *et al.* [16] proposed a poly-Si thermoelectric micro generator, which had an output voltage per area of 22 mV mm^−2^ K^−1^ and a power factor of 4.26 × 10^−4^ μW mm^−2^ K^−2^. In this work, the generator had an output voltage per area of 0.093 mV mm^−2^ K^−1^ and a power factor of 6.4 × 10^−7^ μW mm^−2^ K^−2^. In comparison to the literature, the output voltage and power factor of this work were lower than that of Huesgen *et al.* [7] and Strasser *et al.* [16].

## 5. Conclusion

The thermoelectric micro generator has been implemented using the CMOS-MEMS technique. The micro generator contained 24 thermocouples in series, and the materials of the thermocouples were p-type and n-type polysilicons. The output power of the generator was proportional to the square of the temperature difference between the hot and cold parts in the thermocouples. In order to increase the temperature difference of the thermocouples, the cold part was covered with a silicon dioxide layer to insulate the heat source, and the hot part was suspended and connected to an aluminum plate for increasing heat-receiving. The micro generator needed a post-CMOS process to release the aluminum plate and the hot part of the thermocouples. The post-CMOS process employed an anisotropic dry etching with RIE CHF_3_/O_2_ to remove the oxide sacrificial layer, and then an isotropic dry etching with RIE XeF_2_ was adopted to etch the silicon substrate to release the suspended structures. The advantage of the post-CMOS process was that it was compatible with the commercial CMOS process. Experimental results revealed that the output voltage of the micro generator was about 67 μV at the temperature difference of 1 K.

## Figures and Tables

**Table 1.** Thickness and thermal conductivity of the materials.

**Material**	**Thickness (μm)**	**Thermal conductivity (pW/μm-K)**
Al	0.6	2.36 × 10^8^
SiO_2_	5	1.42 × 10^6^
Polysilicon	0.3	3.2 × 10^7^
